# Apparent Motion Suppresses Responses in Early Visual Cortex: A Population Code Model

**DOI:** 10.1371/journal.pcbi.1005155

**Published:** 2016-10-26

**Authors:** Nathalie Van Humbeeck, Tom Putzeys, Johan Wagemans

**Affiliations:** Laboratory of Experimental Psychology, Department of Brain & Cognition, University of Leuven, Leuven, Belgium; Technische Universitat Chemnitz, GERMANY

## Abstract

Two stimuli alternately presented at different locations can evoke a percept of a stimulus continuously moving between the two locations. The neural mechanism underlying this apparent motion (AM) is thought to be increased activation of primary visual cortex (V1) neurons tuned to locations along the AM path, although evidence remains inconclusive. AM masking, which refers to the reduced detectability of stimuli along the AM path, has been taken as evidence for AM-related V1 activation. AM-induced neural responses are thought to interfere with responses to physical stimuli along the path and as such impair the perception of these stimuli. However, AM masking can also be explained by predictive coding models, predicting that responses to stimuli presented on the AM path are suppressed when they match the spatio-temporal prediction of a stimulus moving along the path. In the present study, we find that AM has a distinct effect on the detection of target gratings, limiting the maximum performance at high contrast levels. This masking is strongest when the target orientation is identical to the orientation of the inducers. We developed a V1-like population code model of early visual processing, based on a standard contrast normalization model. We find that AM-related activation in early visual cortex is too small to either cause masking or to be perceived as motion. Our model instead predicts strong suppression of early sensory responses during AM, consistent with the theoretical framework of predictive coding.

## Author Summary

Two spatially separate stimuli presented in rapid succession often induce the illusory perception of a moving stimulus (apparent motion or AM). Its underlying mechanism is thought to be increased activation in primary visual cortex representing the motion path. Indirect evidence for this account comes from the reduced detectability of stimuli presented along the motion path (AM masking). Here, we developed a computational model of AM-related effects on visual processing in early visual cortex, which predicted a neural activation that is too small to either account for the observed masking or the perception of motion. Instead, our model predicts strong suppression of neural responses to stimuli presented along the motion path, especially when they match the spatio-temporal prediction of a stimulus moving along the path. Our findings support predictive coding models of visual processing, in which higher-level predictions about motion explain away lower-level responses to expected sensory input.

## Introduction

Apparent motion (AM) is a type of illusory motion that can be perceived when two stimuli are presented alternately at two different locations [1]. Under optimal spatial and temporal stimulus conditions, observers can perceive a single stimulus moving continuously along a path between the two locations. Some studies found neurons in primary visual cortex (V1) to respond during AM as if the stimulus was physically present at intermediate locations along the AM path [2, 3]. It has been claimed that humans perceive AM because of these V1 responses, indicating that AM has an early cortical locus [3]. AM-related V1 responses may be the result of feedback from higher visual areas involved in motion (MT/V5) [4–6] or form processing (anterior temporal lobe) [7, 8]. However, evidence for the neural mechanism underlying AM remains inconclusive. Liu et al. [9] failed to find AM-related activity in primary visual cortex during the percept of moving concentric rings. These authors did find increased responses in motion processing areas, suggesting that AM has a late cortical locus. Other studies report a similar lack of activation in V1 [10–12].

Behavioral studies have supported the hypothesis of AM-related activation in V1 by reporting impaired perception of stimuli presented along the path of AM [13, 14]. For instance, Hidaka et al. induced an AM percept using Gabor gratings of a specific orientation. They observed that detectability of a target grating was impaired along the AM path, but only when the orientation of the target matched the orientation of the AM-inducing gratings. This AM masking has been explained by perceptual filling-in at the level of V1. The presentation of the AM inducers evokes responses in a subset of V1 cells, tuned to locations along the AM path and to the visual properties of these inducers. These evoked responses interfere with the responses to actual stimuli along the AM path, thus impairing the perception of these stimuli.

However, AM masking does not necessarily imply V1 activation. An alternative view on AM masking can be provided by a predictive coding account of visual processing. Predictive coding emphasizes the notion of a “predictive brain” generating predictions about incoming information based on the surrounding context [15, 16]. Activation in higher-level visual areas represent the generated prediction based on lower-level input, while lower-level responses represent the mismatch between sensory and predicted input (i.e., prediction error). Predictive signals from higher-level areas are sent back to lower-level areas to reduce prediction error by suppressing sensory signals that can be expected based on the higher-level hypothesis. Presumably, early visual areas receive inhibitory feedback from the motion areas hMT/V5+ [4–6]. Several physiological studies have indeed demonstrated that sensory signals which can be predicted from their surrounding motion context evoke smaller responses in V1 [17–19].

AM stimuli may provide such a predictable motion context. A stimulus presented on the AM path can be considered predictable when (1) its features match those of the apparently moving stimulus and (2) the stimulus appears at a time and place that is consistent with the perceived motion. According to predictive coding models, responses to stimuli presented along the AM path will be suppressed in early visual brain areas such as V1, but only when they are predictable. Using fMRI, Alink et al. [17] confirm this claim, reporting reduced V1 responses to a stimulus during AM only when this stimulus appeared at the expected time and place along the AM path. Interestingly, AM-induced masking seems to be consistent with this kind of suppression. Indeed, Hidaka et al. [13] report that masking of a target grating presented on the AM path only occurs when the target orientation matches that of the apparently moving grating. Presumably, observers perceiving the apparent movement of a horizontal grating expect to see a horizontal but not a vertical grating in the middle of the AM path. V1 responses to a horizontal target grating are therefore predictable and consequently suppressed, while V1 responses to a vertical target grating are unaffected.

In summary, it is at present unclear whether AM masking is the result of V1 activation or suppression. In the present study, we use computational modeling to uncover the actual cause of AM masking, thereby revealing the effects of AM on early visual processing. Similar to Hidaka et al. [13], we used grating inducers to create an AM percept and find masking of a target grating along the AM path. If AM masking is indeed the result of activation in lower-level visual areas such as V1, this masking can be considered to be a special case of pattern masking. In a typical pattern masking study, a target grating has to be detected against a stationary background grating. When the contrast of the background grating is sufficiently high, the target grating is masked. Pattern masking is typically attributed to activation at the level of V1. The background grating evokes a response in V1 cells, and this response can interfere with the response to the target grating. Contrast normalization models provide an excellent account of pattern masking, explaining how activation in low-level stages of visual processing leads to masking [20–22]. If AM indeed induces V1 activation, in line with the early filling-in hypothesis, and if that activation is the sole cause of AM masking, a normalization model should be able to account for this masking.

We developed a V1-like population code model by extending a standard contrast normalization model to include effects of AM. We find that this model cannot account for our results when incorporating only AM-related activation. The amount of activation predicted by the model is too small to be perceived by our observers and does not cause significant masking. Instead, a model incorporating strong suppression of responses to stimuli on the AM path captures the observed masking effects, arguing in favour of predictive coding theory.

## Results

### AM masking

**Fig 1:**
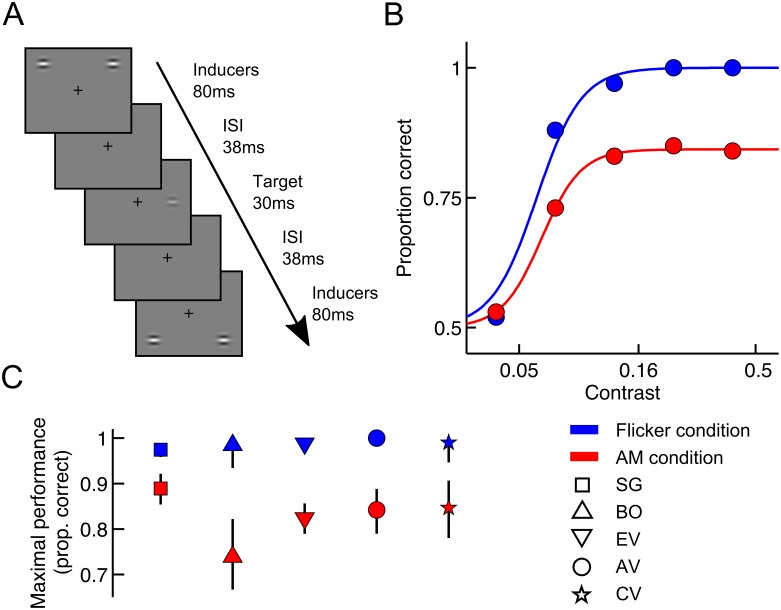
AM induces decreased contrast detection performance. (A) Illustration of part of the trial sequence in the AM condition of the 2AFC task. The figure only shows the fourth sequence of AM during which the target grating was briefly presented. Contrast of the target grating has been increased for illustration purposes. (B) Maximum contrast detection performance of a typical observer (AV) is considerably lower in the AM condition (red) than in the Flicker condition (blue) when the orientation of the target grating and inducers is identical. Full lines depict the best-fitting logistic psychometric function. (C) Maximal detection performance (1 − *λ*) is lower in the AM condition than in the Flicker condition for all observers. Symbols denote different observers. Error bars represent the 95% confidence interval.

We measured the effect of perceiving AM on the detection of a target grating in a spatial two-alternative forced-choice (2AFC) task. In the AM condition, two alternately presented grating stimuli induced a strong percept of AM along a vertical path at both sides of the screen. The target grating was presented either at the left or right side, in the middle of the path. In a Flicker control condition, the two inducers appeared simultaneously, which disrupted the motion percept entirely. Grating contrast and the difference between target and inducer orientation were varied systematically (see Fig 1A for an illustration of part of the trial sequence in the AM condition).

When the target orientation matched the orientation of the inducers, strong masking was observed during AM. Observers failed to reach a high detection performance in the AM condition, even when grating contrast was high. The maximum performance is captured by the psychometric function via 1 − *λ* (see Methods) and was estimated at 83% on average across observers (Fig 1C). The difference in maximum performance between AM and Flicker condition was significant (average *λ* difference = 16%, parametric bootstrap, *p* < 0.05 for all observers after Bonferroni correction). The position of the psychometric function along the contrast axis did not significantly differ between the AM and Flicker condition. Fig 1B shows the data of a typical observer when target orientation was identical to the orientation of the inducers. As all observers displayed similar patterns, we pooled the data across observers (see S1 Text). The pooled data set will be used in the remainder of this study.

**Fig 2:**
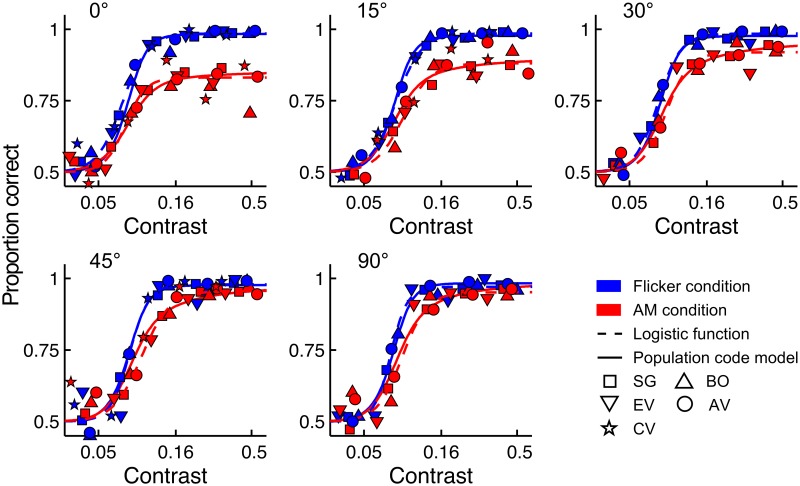
Orientation tuning of AM masking and model fits. The pooled data of the five observers are shown for each orientation level. Red and blue symbols represent the AM and Flicker conditions, respectively. Dashed lines depict the best-fitting logistic psychometric function, while full lines represent the best-fitting contrast normalization model. Symbols denote different observers.

**Fig 3:**
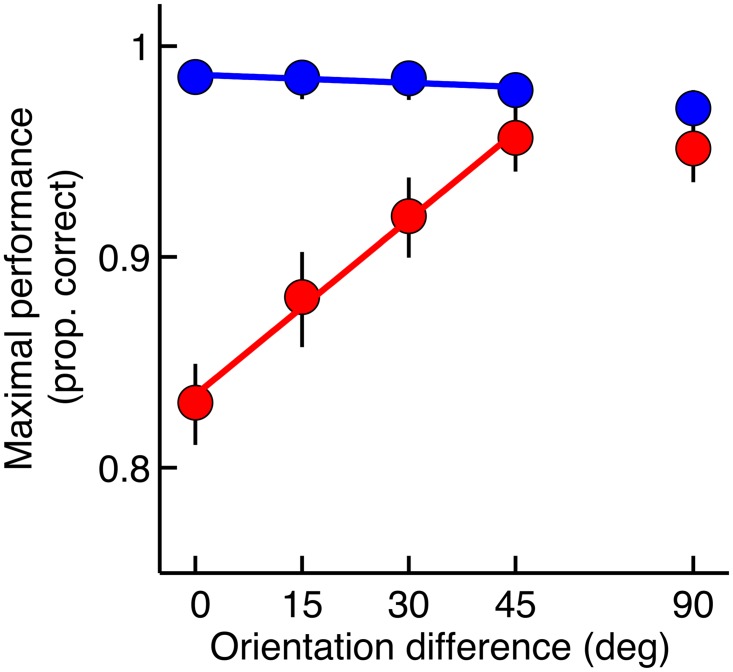
Dependence of maximal performance on orientation of the target grating. Full lines depict regression lines reflecting maximal performance for orientation differences between target and inducers in the 0°–45° range. Error bars denote the 95% confidence interval. When orientation difference increased from 0° to 45°, maximal performance (1 − *λ*) increased significantly in the AM condition (red), while performance remained constant in the Flicker condition (blue). Maximal performance was not affected when increasing the orientation difference from 45° to 90° in either the AM or Flicker condition.

Fig 2 displays the psychometric functions fitted to the pooled data for each orientation level. AM masking appears to be tuned for orientation: the observed masking in the AM condition decreased when the orientation difference between target and inducers increased. This is also evident from Fig 3, which shows the maximum performance level (1 − *λ*) as a function of the orientation difference for the AM and Flicker condition. Maximum performance (1 − *λ*) in the AM condition increased significantly when the difference between target and inducer orientation was increased from 0° to 45° (linear regression slope = 0.0028, parametric bootstrap, *p* < 0.001). In the Flicker condition however, performance was constant in the 0°–45° range (linear regression slope = -0.0001, parametric bootstrap, *p* = 0.865). Increasing the orientation difference from 45° to 90° did not affect maximal performance in either the AM or Flicker condition (pairwise comparison, parametric bootstrap, *p* > 0.05 for both conditions). Maximum performance was significantly higher in the Flicker condition than in the AM condition for all orientation levels (pairwise comparison, parametric bootstrap, *p* < 0.05 after Bonferroni correction). The steepness of the psychometric function controlled by *s* did not differ between the AM and Flicker condition for any of the orientation levels (average difference = 0.0025, parametric bootstrap, *p* > 0.05 for all observers).

### Population code model of AM-induced effects

**Fig 4:**
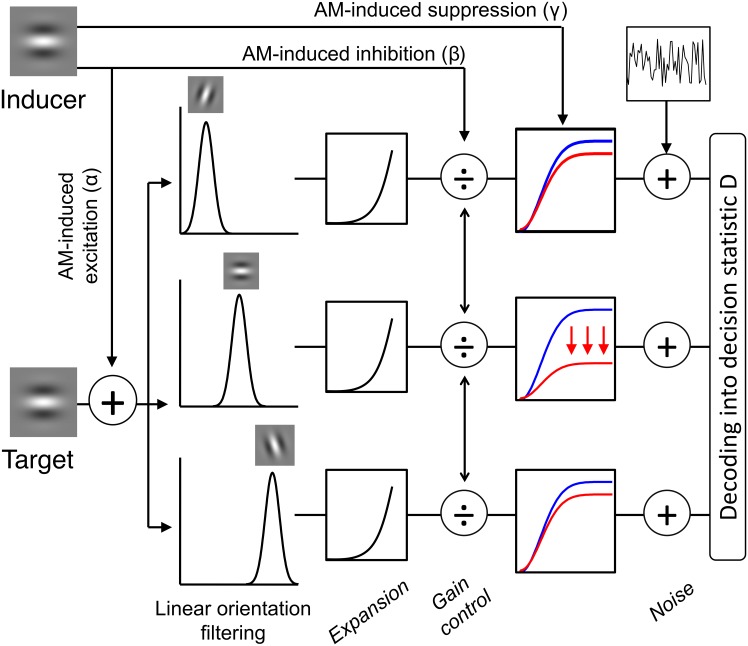
Schematic overview of possible AM-induced effects in the contrast normalization model. Following the standard contrast normalization model, the target grating in our task is encoded by a population of V1-like neurons tuned to orientation, which are subject to response acceleration and divisive inhibition. This standard model is extended by including the effects of AM, which can modify the encoding of gratings in our model in three ways. (1) AM can excite linear receptive fields sensitive to the orientation of the inducers controlled by *α*, evoking responses as if the inducer was physically present at the target location (as during real motion). This would correspond to a “filling-in” process along the AM path. (2) AM can induce divisive normalization via *β* by exciting neurons in the gain control pool tuned to the orientation of the inducers. A similar divisive normalization signal would be observed when the inducer would be positioned at the target location. As such, AM-induced inhibition is also in accordance with a “filling-in” account of AM. (3) AM can scale down the contrast response functions due to the suppressive effect exerted by neurons tuned to the inducers’ orientation via *γ*.

We developed a V1-like population code model (see Methods), based on the contrast normalization model [20, 22–24], to incorporate AM-induced effects on low-level visual processing. In our model, the effects of AM on the encoding of a target grating can potentially occur in three different ways (for a schematic overview, see Fig 4). First, AM can cause excitation by “filling-in” activation along the AM path. More specifically, AM can induce a response in neurons sensitive to the inducer orientation. Second, AM can inhibit responses in the neural population by exciting the gain control pool and hence causing normalization. It should be noted that the excitatory and inhibitory AM effects mimic the excitation and inhibition that would be observed in the case of real motion. In other words, the neural population responds “as if” the inducer was actually moving along the AM path, in accordance with a “filling-in” account of AM. A third way AM can affect the population response is by reducing the response gain of neurons sensitive to the inducer orientation. In this case, the maximum response is reduced as the entire contrast response function is rescaled to lower response rates. We consider such a suppressive effect because of our finding that AM reduces maximum performance and that a rescaled psychometric function is required to capture this observation. Note that, in this study, AM-induced effects refer to effects caused by the inducers during the AM sequence, presumably via feedback from motion areas, and not necessarily caused by the conscious percept of AM.

### Evaluation of model fit

Our model accurately captures the observed AM masking and its dependence on the orientation difference between the target and inducers (see Fig 2). We compared the AIC of this model with the AIC of the best-fitting logistic psychometric function model which depends on fewer theoretical constraints. The AIC of our model was not significantly higher than this model (AIC difference = 12.74, parametric bootstrap, *p* = 0.27), meaning that our population code model provided a good fit in comparison to a highly flexible psychometric function model.

### Response suppression during AM

Maximum-likelihood fitting provided the optimal parameter estimates of our model. Most estimates are well within the range of values reported in physiological V1 single-cell recording studies in monkeys or cats. The semi-saturation contrast was estimated at 9.65%. A similar value has been reported in a physiologically plausible population code model of human contrast processing [25]. The spontaneous background activity equalled 4.55% of the maximal response, matching reports of Geisler and Albrecht [26]. The concentration parameter *k*_*exc*_, controlling the bandwidth of excitatory (Von Mises) orientation tuning functions, was estimated at 1.35 (95%*CI* = [0.93, 3.52]). This corresponds to an orientation bandwidth at half-height of 41.92° (95%*CI* = [25.62°, 51.17°]), in agreement with observed bandwidths of V1 orientation tuning functions [27]. The response exponent *p*, determining the degree of non-linear response expansion, was estimated at 5.52. This is a relatively high value in comparison with physiological findings (see Methods and Discussion) [28]. Our efficiency parameter *ϵ* was estimated at 67%. As noted previously, the exact value of the efficiency parameter may reflect a wide range of possible factors which merely affect absolute performance and do not mediate AM-specific masking.

**Fig 5:**
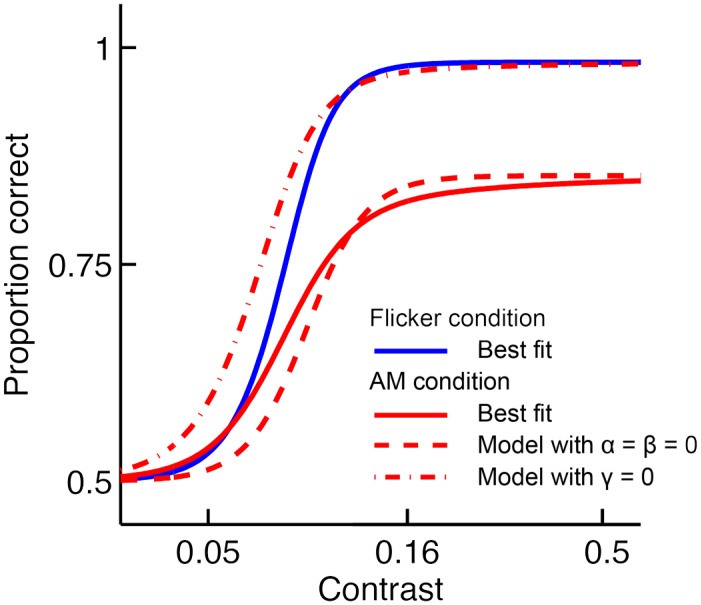
Comparison of contrast normalization models in capturing AM masking. The pooled data of five observers are shown for the condition in which the orientation of target and inducers is identical. The predictions of the best-fitting contrast normalization model including AM-induced effects is indicated by the full red line (AM condition) and full blue line (Flicker condition). The dashed line shows the model prediction for the AM condition when excitation and divisive inhibition effects are removed after fitting (*α* = *β* = 0). The dash-dotted line represents the prediction when the suppression effect is set to zero after fitting (*γ* = 0). In contrast to the model without excitation and divisive inhibition, the model without suppression fails to account for the observed masking at high contrast levels. In addition, this model predicts facilitation of contrast detection at low contrast levels, which is not supported by the data. All three models predict the same performance for the Flicker condition, as all parameters controlling AM effects (*α*, *β* and *γ*) are set to zero for this condition.

Possible effects underlying AM in our model are excitation, inhibition through divisive normalization and response gain suppression, controlled by *α*, *β*, and *γ*, respectively. AM induces significant excitation: *α* was estimated at 1.17% (95%*CI* = [0.62%, 1.68%]). AM also causes significant inhibition, as estimated by *β* which equalled 1.51% (95%*CI* = [1.20%, 2.06%]). Importantly, AM-induced excitation and inhibition have only a limited effect on the contrast response functions and, consequently, on detection performance. To demonstrate the contribution of excitation and inhibition to masking, Fig 5 shows the predictions of the model for the case in which *α* and *β* were set to zero after model fitting. It can be seen that the model still predicts a considerable amount of masking when AM-induced excitation and inhibition are removed. The reason is that masking is mainly caused by response gain suppression in our model. *γ* equalled 64.4% (95%*CI* = [56.4%, 76.5%]), indicating that the contrast response function in the presence of AM is scaled down by a factor of (100 − 64.4)% = 35.6%. Fig 5 shows the predictions of the best fitting model in which *γ* was set to zero after fitting. Not only is masking significantly reduced at high contrast levels, at low contrast levels the model predicts facilitation. In other words, detection performance is predicted to be better in the AM condition compared to the Flicker condition when target grating contrast is low. This facilitation effect was not present in the data. In agreement with the predictions of the logistic psychometric function model, the population code model predicts that performance in the AM condition is always lower than in the Flicker condition, also at lower contrast levels. To further test whether a model including only excitation and inhibition could account for our results, we refitted the model without suppression (*γ* was constrained to zero before fitting). The AIC of this model was significantly worse than the AIC of our full model including suppression (AIC difference = 212.62, parametric bootstrap, *p* < 0.001), indicating that the latter provided a better fit to the data.

### AM-induced effects are tuned to orientation

The effects induced by AM in our model are selective to orientation. More specifically, the size of the AM effects on a neuron decreases as the difference between the neuron’s preferred orientation and the inducer orientation increases. For AM-induced excitation as well as response gain suppression, the tuning functions have an orientation bandwidth equal to the bandwidth of the neurons’ excitatory receptive fields, as evident from the fact that the concentration parameters *k*_*exc*_ and *k*_*exc*,*AM*_ are equal. Their value of 1.35 (95%*CI* = [0.81, 2.00]) corresponds to an orientation bandwidth at half-height of 41.92° (95%*CI* = [54.98°, 34.22°]). AM thus only affects neurons sensitive to the inducer orientation, with an orientation precision that matches the orientation selectivity of typical V1 cells [27, 29]. The bandwidth concentration parameter of the tuning function for the AM-induced inhibition was estimated at 0.001. This small value implies that inhibition is not tuned for the inducer orientation and is consistent with a broadly tuned gain control pool. To examine whether a model which allows no orientation tuning of the AM-evoked effects could also account for our data, we compared the AIC of our model with the AIC of a model in which *k*_*exc*,*AM*_ and *k*_*inh*,*AM*_ are fixed at 0.001 before fitting. Our full model was evaluated significantly better than the model containing no tuning of AM effects (AIC difference = 136.55, parametric bootstrap, *p* < 0.001).

### Early excitation cannot be considered as perceptual filling-in

The small level of AM-induced excitation can be seen as filling-in of activation along the AM path, as mentioned earlier, but to what extent can it be considered as *perceptual* filling-in? To answer this question, we used our population code model to predict the detectability of a grating evoking the same level of activation as induced by AM. This is a grating with contrast equal to *α* = 1.17%, following from the fact that the estimated values of *k*_*exc*_ and *k*_*exc*,*AM*_ are equal (see Eq (10) in Methods section). Our best-fitting model predicts that even in the Flicker condition, in which the AM-induced suppression is absent, such a grating cannot be detected (detection performance would equal 50%).

### Eye movements are not the cause of AM masking

Note that observers were instructed not to move their eyes and maintain fixation on the centre of the screen throughout the entire trial duration. It is however possible that observers did not follow these instructions and that the observed AM-induced effects can be explained by systematic differences in eye movement patterns between the AM and Flicker condition. We therefore conducted a control experiment in which three observers performed the contrast detection task while eye movements were recorded (see S2 Text).

Observers were found to be highly successful in maintaining fixation on the cross in the centre of the screen. For one observer, significant differences in fixation behavior were observed between the Flicker and AM condition. These small differences however could not account for the observed AM-induced masking. Our population code model was able to account for this masking effect and parameter estimates were highly similar compared to the estimates obtained in the main experiment. The modeling results show that all AM-induced effects found in the main experiment were present and equally strong in the control experiment. In summary, eye movements do not seem to play a role in AM masking nor in the underlying AM-induced effects on early sensory responses.

## Discussion

### Early response suppression instead of perceptual filling-in during AM

In the present study, we examined the underlying neural effects of AM on low-level visual processing. To this end, we investigated how AM influences the detection of grating stimuli presented along the AM path. We found that AM impairs the detection of a target presented on the AM path when the target’s orientation matches the inducers’ orientation. A previous study reporting AM masking measured detection performance at a single stimulus intensity level [13]. We evaluated detection performance across a wide range of grating contrast levels in order to obtain full psychometric functions. This allowed us to discover that perceiving AM imposes an upper bound on performance, which cannot be exceeded by raising the contrast level of the target grating.

We applied a V1-like population code model, based on contrast normalization, and found that this model provides an excellent account of the data, predicting the AM-induced upper bound on performance. Importantly, the model reveals that AM masking is not caused by excitation but by suppression of responses to stimuli along the AM path, through a reduction in response gain. In fact, when this suppressive effect is removed from our model, masking disappears and the model even predicts facilitation. This is related to the well-known pedestal effect, which refers to the observation that the presence of a background grating can facilitate the detection of a superimposed target grating at low background contrasts. The pedestal effect has been attributed to the fact that the background grating evokes a small level of activation in V1 [21]. Due to this activation, target detection operates on a steeper part of the V1 contrast response functions, leading to larger differential responses. According to our model, the AM-induced activation also acts as a pedestal, improving detection performance. This facilitation effect does not show up in the data due to the strong suppressive effect of AM. The results of a control experiment rule out the possibility that eye movements were responsible for the observed masking or AM effects on early sensory responses.

Previous studies have claimed that human observers perceive motion when viewing an AM display because of filling-in of V1 activation along the AM path [3, 13]. This perceptual filling-in would imply that AM is (at least partly) represented at the level of V1. Our results suggest that AM-induced activation is too small to be perceptually relevant. A grating on the AM path evoking the same level of excitation as the level of AM-induced activation predicted by our model would not be detectable. Consequently, AM would most likely not be perceived if it was only represented in this small amount of V1 activation. The AM-evoked excitation can thus hardly be considered as an instance of perceptual filling-in. This result provides strong support for the hypothesis that the AM percept is due to activation at a later processing stage.

However, it is at present not clear which higher-level brain areas are involved in the representation of AM. Some neuroimaging studies indicate that the motion area hMT/V5+ plays an important role in the perception of AM [5, 6, 9, 10]. This area contains neurons selective to motion direction with large receptive fields, which seems necessary for an AM percept occurring over a relatively large distance (i.e., 8° in our study). Such type of perceived motion has been contrasted with short-range AM (i.e., motion occurring over a limited spatial and temporal range), which has been hypothesized to be represented at lower levels of the visual processing hierarchy [30, 31]. However, single-cell recording studies in nonhuman primates have failed to find a representation of long-range motion in MT [32–34]. Instead, MT neurons responded only to short-range motion even when long-range motion was perceptually dominant. Moreover, other findings suggest that the visual ventral pathway, which is important for shape processing, is involved in the representation of AM [7, 8]. Irrespective of where AM is represented in the brain, our results suggest that neurons responding to AM will activate a mechanism that ultimately leads to response suppression in early visual areas.

The response gain reduction predicted by our model was strongest when the target matches the orientation of the motion-inducing stimuli. This orientation tuning of AM-induced suppression is implemented in the model by rescaling the effects of AM on a given neuron with the selectivity of that neuron to the inducers. AM thus only affects neurons that are selective for the inducers’ orientation. It should be noted that, due to our stimulus design, detection performance for a horizontal target grating was always worse than for a vertical grating in the AM condition. The degraded detection performance for a horizontal grating in the AM condition could be due to a difference between the Flicker and AM condition in preferred direction of eye movements. The control experiment ruled out this alternative explanation.

### AM-induced effects occur in the early visual cortex

AM most likely modulates sensory responses in the early visual cortex. Evidence for this claim comes from the fact that the response properties of the neurons assumed in our model’s encoding front-end are consistent with those of V1 neurons. All parameter estimates specifying the front-end match estimates reported by physiological studies of V1, with the exception of the response exponent *p*. The value of *p* was estimated at 5.52, which is higher than the value of 2 typically observed in V1. The large response exponent had to be assumed in the model to account for the large steepness of the psychometric functions. As such, the response exponent estimate does not only reflect the degree of V1 response acceleration but can also capture other factors contributing to psychometric function steepness which were not incorporated in our model to keep computations tractable (see Methods). Spatial uncertainty, for instance, has been found to increase the steepness of the psychometric function [35]. Notably, spatial uncertainty is higher in the periphery compared to the fovea [36, 37]. In our experiment, the target was positioned relatively far in the periphery (i.e., 10°), making it possible that uncertainty contributed to the steepness of the psychometric functions. Importantly, it is unlikely that a possible increase in spatial uncertainty played any role in AM, as the slope of the psychometric functions did not differ between the AM and Flicker condition. A reduction in maximum performance, which is the key aspect of AM masking, has not been linked to spatial uncertainty [36]. The large value of the response exponent is thus not inconsistent with the claim that the encoding front-end captures the responses of V1 neurons.

Although a population code model of V1 is able to capture our results, we cannot rule out the possibility that AM-induced effects occur in other areas of the early visual cortex. Indeed, while multiple physiological [38, 39] as well as psychophysical [21, 23, 24, 40] studies have linked changes in contrast detection and discrimination performance to response changes in a V1 population of neurons, activity in areas V2 and V3 has also been found to correlate with decisions in contrast detection tasks [41]. Even the majority of V4 neurons show monotonic sigmoidal contrast responses that can be characterised using parameter values largely similar to the ones we used [42], especially in case of short stimulus presentation durations [43].

Nevertheless, the orientation tuning bandwidth estimates found in our study do argue against AM-induced suppression occurring in V4 and higher-level areas. The confidence interval of the *k*_*exc*_ parameter has a relatively low upper bound of approximately 50°. In other words, our detection task mainly involved narrowly-tuned neurons, i.e., with a bandwidth (at half height) lower than 50°. While this value matches the estimates found for V1 [27] and V2 neurons [44], the bandwidths found in higher-level areas are considerably larger [29]. David, Hayden, and Gallant [45], for instance, explicitly compare the orientation tuning bandwidth of individual V1 [27, 46] and V4 [47] neurons. They find that the majority of V4 cells have a bandwidth larger than 60°, with a median as large as 74.4°. The median for V1 cells equalled 43.7°, with a considerable amount of bandwidth values being smaller than 60° and, as such, part of the confidence interval estimated for our *k*_*exc*_ parameter. Even though the distribution of tuning bandwidths observed in V1 is known to be broad, the average bandwidth is lower compared to the average bandwidth observed in higher visual areas. It is this average bandwidth that is captured by our model’s *k*_*exc*_ parameter. The bandwidth estimate of the orientation tuning functions capturing AM-induced effects also matches the average bandwidth of V1 neurons, which again suggests a V1 locus of AM effects.

### Response suppression can be explained by predictive coding theory

Our computational modeling results only provide a description of the suppressive effects of AM on responses in early visual cortex. Although such a description is interesting and useful in its own right, it does not explain why AM induces suppression. An explanation can be provided by predictive coding theory, which assumes that responses in low-level visual areas signaling prediction error are suppressed when they are consistent with the higher-level prediction of motion by a single stimulus along the AM path. Such an explanation is in accordance with other studies reporting reduced V1 activation for local features that fit their surrounding context [17–19, 48, 49]. For instance, Alink et al. [17] found that the predictability of stimuli in their surrounding AM context leads to reduced activation in V1. However, since the authors did not include a control Flicker condition, responses to a predictable stimulus in the context of AM could not be compared to those evoked by an unpredictable stimulus in the absence of AM. Our model predicts that responses to a physically present stimulus in the AM condition would be lower than those to a stimulus in the Flicker condition and that this suppression of responses to predictable sensory input is the main cause of AM masking. It should be noted that this is different from the paradigm used by Muckli et al. [3], in which no stimulus was present along the AM path and suppression of responses to such a stimulus could consequently not be measured.

Stimulus predictability is a key element of the predictive coding framework, which claims that responses to a stimulus are suppressed only when the occurrence of those stimuli is predictable or expected. It should be noted that our manipulation of target grating orientation can be interpreted as a manipulation of the predictability of the target stimulus. Arguably, when viewing the apparent movement of a horizontal grating, observers may expect a horizontal target grating appearing in the middle of the AM path. The percept of this grating can be interpreted as being part of the motion percept: the apparently-moving horizontal grating is expected to pass by the middle of the AM path at the exact moment the target grating is presented. However, suppose we present a vertical target grating. This grating is presumably unexpected, as observers expect to see the horizontal grating passing by the middle of the AM path at the time the vertical grating is presented. By manipulating the target grating orientation in the present study, we induced both predictable as well as unpredictable responses in early visual cortex. We find that mainly the predictable responses, i.e., responses of neurons tuned to the predictable horizontal grating orientation are suppressed. As the orientation of the target grating deviates more from horizontal, the neurons responding to that grating are suppressed less. This finding supports the claim of predictive coding models that stimulus predictability leads to suppression.

Suppression through response gain reduction is a multiplicative effect that can be implemented in a predictive coding framework [16]. Multiplicative or proportional scaling of responses of lower-level neurons has been reported by multiple studies [42, 50, 51]. In addition, it has been found that GABA_*A*_ inhibition controls response gain in V1, without affecting contrast gain [52]. It is possible that GABA_*A*_ levels are selectively increased during AM. Indeed, GABA-mediated cortical inhibition has been linked to predictive coding in a recent study [53].

Although the predictive coding framework provides a functional explanation of our results, any other theory that predicts orientation-tuned AM-induced suppression can in principle account for these results. The descriptive nature of our population code model prevents us from providing direct evidence in favor of one particular theoretical framework. Our main contribution lies in the fact that we managed to use our descriptive model to reject the conclusions of multiple previous studies by uncovering a suppressive rather than facilitatory mechanism underlying AM. Any future theory of AM will have to account for this mechanism.

### Nature of AM-induced suppression

In our model, V1 responses are inhibited during AM in two distinct ways, namely reduction in response gain and contrast gain (i.e., contrast sensitivity) [50, 51, 54]. Contrast response functions were found to be rescaled to lower response rates during AM, indicating a decrease in response gain. At the same time, a gain control mechanism is activated during AM, which shifts the contrast response function to higher contrasts, thereby lowering contrast sensitivity. Importantly, the response gain effect is much larger than the change in contrast sensitivity. In addition, the change in response gain is much more narrowly tuned for the inducers’ orientation. This seems to suggest that the two forms of inhibition reflect different neural mechanisms. We believe that the response gain reduction is a direct consequence of AM because it is narrowly tuned. The change in contrast sensitivity seems to be a more indirect effect in that it may result from the activation of the gain control mechanism by the small AM-induced excitation. Indeed, it has often been found that the gain control mechanism is broadly tuned for orientation [55]. Presumably, inhibition by neurons that are not sensitive to the inducers’ orientation results in weak AM masking to be still present in our data when the target and inducers are orthogonal. We incorporated response gain reduction in our model as a linear rescaling of the contrast response function similar to previous studies [42, 50, 51], but other implementations may be possible. For instance, Rosenberg et al. implement a decrease in response gain via divisive normalization [56]. More specifically, they assume an amplification of the normalization signal, which causes the contrast response functions to scale to lower response rates. This non-linear rescaling approximates the linear rescaling implemented in our model to a large degree. It is therefore possible that a model incorporating changes in divisive normalization during AM may account for the observed suppression. However, such a model would also have to predict the contrast gain reduction observed during AM in the present study. A contrast gain decrease corresponds to an additive increase of the normalization signal, whereas response gain reduction is realized through a multiplicative increase. Although it is possible that changes in the normalization mechanism are compound, consisting of both additive and multiplicative effects, previous studies have mainly reported additive changes leading to a change in contrast gain [20]. Our model therefore only incorporates additive changes in normalization during AM.

### Robustness of the population code model

A highly similar version of our population code model, consisting of a physiologically plausible encoding front-end and a simple linear decoder, has been used previously by Putzeys et al. [24]. For the model to be considered robust, the parameter estimates obtained in the current study should be consistent with those reported by Putzeys et al. [24]. It should be noted that some of the parameters are poorly constrained by the data and their exact values are not critical. Differences in these values between current and previous studies should not be taken as evidence against the model. For instance, the spontaneous discharge rate was estimated at 4.55% in our study, but was poorly constrained in the Putzeys et al. study [24]. The proportionality constant was poorly constrained in both studies. Only two of the well-constrained parameters estimates were different compared to the Putzeys et al. study [24]. Firstly, the response exponent *p* was estimated at 5.52, which is higher than the physiologically plausible value of 2 used by Putzeys et al. Secondly, the estimated semi-saturation contrast *c*_50_ equalled 9.65% in the present study while Putzeys et al. reported a value of only 3%.

As discussed earlier, we attribute the large response exponent to the high spatial uncertainty in the periphery compared to the fovea [36, 37]. While we presented targets in the periphery, Putzeys et al. [24] used a temporal 2AFC task with all stimuli being presented in the fovea. It is therefore likely that the amount of spatial uncertainty was lower in the latter study, which led to less steep psychometric functions and, consequently, a lower response exponent.

The difference between foveal and peripheral grating presentation may also account for the difference in semi-saturation contrast. It is known that contrast sensitivity is lower in the periphery [57]. The contrast sensitivity of a V1 neuron is determined by its semi-saturation contrast value. The high estimate of this value may simply reflect the low peripheral contrast sensitivity. Furthermore, it has been suggested that spatial attention can increase the contrast sensitivity of V1 neurons by lowering their semi-saturation contrast value [40]. In the spatial 2AFC task of the current study, spatial attention had to be divided over two regions in the periphery, while in the temporal 2AFC task of Putzeys et al. [24], attention could be fully directed to a single foveal region. This higher level of attention may have lowered the semi-saturation contrast.

### Possible extra-classical receptive field effects

A possible issue concerns extra-classical receptive field effects operating at the level of V1, such as surround facilitation and suppression [58–60]. These effects involve an increase (surround facilitation) or decrease (surround suppression) of the response gain of individual classical receptive fields by stimuli presented in their spatial surround [61, 62]. Extra-classical receptive field effects may arise due to the size of our stimuli. The target grating stimulus to be detected in our task is relatively large, with a length and width equalling 4.5°. As such, it is likely that neurons with their classical receptive fields tuned to the centre of this stimulus are affected to some extent by the target stimulus contrast presented in their extra-classical surround [63]. Gain modulations of these neurons will occur in both the AM and Flicker condition and can therefore not explain AM-induced suppression. Such response gain changes will only lower overall observer performance, which can be captured by a reduction of our model’s efficiency parameter.

A second possible extra-classical receptive field effect may stem from the fact that, in the Flicker condition, the target grating is presented together with two inducers, while in the AM condition, the target grating is surrounded by maximally one inducer at any given time. One could argue that the inducers cause surround suppression or facilitation in the neurons responsible for detecting the target grating. This would mean that AM-induced effects are an instance of the traditional extra-classical receptive field effects as discussed in, e.g., Henry et al. [59]. However, in that case, we would expect these effects to be larger in the Flicker condition than in the AM condition as the former condition involves more simultaneous surround stimulation. In other words, we would expect facilitation or suppression in the Flicker condition. Rather, we clearly observed that performance is affected in the AM condition instead. Indeed, maximal performance is strongly limited in the AM condition. Such a performance limit is not observed in a typical “baseline” contrast detection task in the absence of inducers. The psychometric functions found in the Flicker condition, on the other hand, do resemble the functions found for standard baseline contrast detection. This suggests that it is the specific spatio-temporal pattern of inducer presentation in the AM condition that causes both the percept of AM as well as response suppression. This suppression can in itself be seen as a dynamic extra-classical receptive field effect, but one that is different from the more traditional extra-classical receptive field effects.

### AM-induced masking cannot be explained by attention

When considering the effect of *spatial* attention, one possibility is that the target grating location is attended less in the AM condition compared to the Flicker condition. The inducers in the Flicker condition may both attract attention, resulting in a locus of attention on a spatial region which includes the target grating. In the AM condition, on the other hand, each inducer appears separately. This may cause observers to alternately focus on the two inducer locations, thereby neglecting the location of the target grating. Recall that our model revealed that AM-induced masking is mainly due to suppression of neurons tuned to the target location. To judge whether spatial attention to one of the inducers can lead to such suppression, one has to consider the scale at which spatial attention operates. Various neurophysiological studies have found that spatial attention to a specific location can indeed lead to response suppression of neurons in early visual areas. However, suppression is limited to neurons tuned to locations in the immediate vicinity of the attended location [64, 65]. Psychophysical studies support this finding [66, 67]. In our experiment, the target is presented at a distance of 4° from the inducers. At such a distance from the locus of attention, suppression is not observed [64, 65, 67]. Consequently, we do not consider spatial attention as a plausible explanation of AM-induced effects.

Likewise, *feature-based* attention cannot account for our results. This form of attention has been found to enhance the processing of attended visual features at the expense of features that are not attended. In our task, the relevant visual feature is arguably grating orientation. However, we found the detectability of the target grating to be *impaired* when its orientation was close to the horizontal inducer orientation. This finding cannot be attributed to feature-based attention improving the detectability of more vertical orientations at the expense of horizontal orientations. If that would indeed be the case, one would expect improved detectability at vertical orientations, which was not observed in our study.

### Conclusion

In the present study, we made three important contributions to the understanding of AM. First, we discovered a central but hitherto unnoticed aspect of AM masking, namely the upper bound on detection performance. Second, we identified orientation-tuned suppression of responses in early visual cortex as the major cause of AM masking. This suppression may be explained by predictive coding models of cortical processing, proposing that higher-level predictions of motion are generated which “explain away” lower-level responses to expected sensory input. Third, we concluded that perceptual filling-in of the motion path does not occur at an early stage of visual processing. Further research is needed to determine exactly where and how AM is implemented in the brain and whether predictive coding theory can fully account for AM-induced response suppression.

## Methods

### Subjects

Five observers (AV, BO, CV, EV, and SG) participated in the experiment. All were naive to the purpose of the study and had normal or corrected-to-normal vision (age range 20–23). The study was approved by the Social and Societal Ethics Committee of the University of Leuven. Written consent was obtained for all participants before the start of the experiment. Observers were paid 8 euros an hour for participating. A block of 50 practice trials were conducted to familiarize subjects with the stimuli and task. All subjects reported having no difficulty in perceiving the AM sequences.

### Apparatus

Stimuli were generated and presented using a ViSaGe stimulus generator (Cambridge Research Systems, Cambridge, England) controlled by MATLAB (MathWorks, Natick, US). A linearized ViewSonic G90fB monitor (ViewSonic, California, USA) was used to display the stimuli. The monitor had a spatial resolution of 1024 x 768 pixels and operated at a refresh rate of 118 Hz, with 8-bit luminance precision for all contrast levels used in the study. Participants were seated in a darkened room with their heads supported by a chin rest, at a viewing distance of 60 cm (corresponding to a pixel size of 0.0315° of visual angle). The mean background luminance of the screen was equal to 72.5 cd/m^2^. Participants’ responses were registered by means of a Cedrus response box (RB-530, Cambridge Research Systems).

### Eye movement recording

A video-based infrared eye tracker was used (EyeLink 1000, desktop version, SR Research Ltd., Ontario, Canada) in a control experiment to ensure that observers’ eye positions remained fixated on the fixation cross in the centre of the screen during the course of a trial (see S2 Text). Movements of the right eye were tracked with a sampling frequency of 1000 Hz. The default settings of the Eyelink software were employed to detect saccades, namely a velocity threshold of 30°/*s* and an acceleration threshold of 8000°/*s*^2^. A calibration procedure was performed at the start of the experiment and was repeated at regular times during the experiment. During the calibration procedure, participants were asked to follow a dot presented at each of nine locations on the screen. This procedure was repeated until the positions of the recorded fixations were aligned on a three by three rectangular grid. At the beginning of each trial, observers were instructed to fixate a cross in the centre of the screen. This cross was presented until the experimenter pressed the space bar, which triggered correction for drifts in recorded eye positions due to small head movements. If deviation between eye position and the central cross was larger than 2°, the eye tracker was recalibrated. Eye movement recording was manually interrupted by the experiment as soon as the observers made a response.

### Stimuli

All stimuli used in the experiment were Gabor patches, created by multiplying a cosine grating with a 2D Gaussian envelope (SD = 0.75°). The spatial frequency of all gratings was 1.5 cycles per degree. Stimuli were displayed on a gray background (Michelson contrast of 50%). Both AM- and Flicker-inducing stimuli had a Michelson contrast of 100%, while the contrast levels of the target stimulus ranged from 4% to 40% Michelson contrast. Target orientation equalled 0° (horizontal), 15°, 30°, 45° or 90° (vertical). The orientation of the inducers was 0°. The target grating was presented at 10° eccentricity either left or right from a fixation cross (0.76° x 0.76°). Two pairs of inducers were used, eliciting either an AM or Flicker percept both at the right and left side of the screen. Within each pair, the inducers were vertically separated by 8° and the target stimulus was positioned exactly in between one of these pairs, at a distance of 4° from each inducer stimulus.

### Detection task

Observers were instructed to detect a target stimulus appearing either at the left or right of a fixation cross in a spatial two-alternative forced-choice (2AFC) task. At the beginning of the trial, a fixation cross was shown for 500 ms. This fixation cross remained on the screen for the entire trial duration and observers were asked to maintain fixation on it. The inducers were then presented for a duration of 80 ms alternately at the top and bottom position at both sides of the screen with an inter-stimulus interval of 106 ms. This AM sequence was repeated four times to induce a strong percept of stimuli moving back and forth. The target was flashed briefly for 30.8 ms during the fourth AM sequence, 38 ms after the presentation of the inducer at the top position and at an intermediate position in the interstimulus interval. At the end of the trial, observers were asked to indicate at which side of the screen the target appeared. No limits were placed on the allowed reaction time. Auditory feedback was provided after each trial. In the Flicker condition, the procedure was identical, except that the inducers simultaneously appeared at the top and bottom. This disrupted the percept of motion completely, while controlling for masking effects resulting from the presence of the inducers [68]. Fig 1A shows an example of part of the stimulus sequence in the AM condition in which the orientation of the target and inducers are the same.

The experiment consisted of blocks of 50 trials, in which contrast (5), orientation (5) and condition (2) levels were randomised. Each subject completed at least 50 trials for each combination of these levels. Due to time constraints, subject CV only completed three of the orientation levels, namely 0°, 15°, and 45°. Psychometric functions were fitted to the individual and pooled data, relating target grating contrast to proportion correct responses. The form of our psychometric function is given by:

$$\psi \left(c;c_{m},s,\lambda \right)=0.5+\left(0.5-\lambda \right)F\left(c;c_{m},s\right)$$ (1)

where *c* denotes the contrast levels of the target grating and *F* is a sigmoidal logistic function of *c* ranging from 0 to 1 [69]:

$$F=\frac{1}{1+e^{(c_{m}-c)/s}}$$ (2)

in which *c*_*m*_ equals the midpoint contrast and *s* determines the steepness of the function. *λ* controls the upper bound of the psychometric function, as *ψ* ranges from 50% to a maximum of 1 − *λ*. Note that *λ* is often considered a lapse rate parameter, reflecting the amount of stimulus-independent errors made by the observer [69]. However, *λ* is estimated here for both the AM and Flicker condition and for each orientation of the target. Hence, *λ* should not be interpreted as a lapse rate, as it will be evident from our results that *λ* is highly dependent on these stimulus conditions. Psychometric functions were fitted using a maximum-likelihood fitting procedure [69]. A parametric Monte-Carlo bootstrap procedure involving 10000 samples provided the distributions of the deviance statistic used to assess goodness-of-fit, as well as the confidence intervals for the parameter estimates [70]. It should be noted that the goodness-of-fit of all psychometric functions fitted in the present study was acceptable (parametric bootstrap, *p* > 0.05 after Bonferroni correction).

### Population code model

#### Encoding stage

Gratings in our task are encoded by a population of V1-like neurons. Each neuron in the population is characterized by a linear excitatory receptive field tuned to orientation [71]. The response of this receptive field to a grating of contrast *c* and orientation *θ* is provided by:

$$L_{i}\left(c,\theta \right)=c*f_{i}\left(\theta \right)$$ (3)

where *f* is a von Mises orientation tuning function [72] rescaled to obtain a maximum of one at the preferred orientation ${\hat{\theta }}_{i}$, irrespective of the bandwidth of the function:

$$f_{i}\left(\theta \right)=\frac{e^{k_{exc}\left(2cos\left(2\left(\theta -{\hat{\theta }}_{i}\right)\right)-1\right)}}{e^{k_{exc}}}$$ (4)

The bandwidth is controlled by the concentration parameter *k*_*exc*_. Note that we implicitly assume that each neuron in the population is spatially tuned to the location of the target grating in the middle of the AM path. Given the large spatial separation of the target and inducers, the neurons in our population are further assumed to operate independently from neurons tuned to the inducer locations. Hence, in our model, neurons tuned to other spatial locations do not play a role in the detection of the target.

The linear response *L*_*i*_(*c*, *θ*) is raised to an exponent *p* to introduce an accelerating non-linearity [28], and divided by a normalization term according to the Naka-Rushton equation [73, 74]:

$$R_{i}\left(c,\theta \right)=\frac{L_{i}{\left(c,\theta \right)}^{p}}{{c_{50}}^{p}+G_{i}{\left(c,\theta \right)}^{p}}$$ (5)

*G*_*i*_(*c*, *θ*) is the normalization signal, i.e., the linear response of a divisive inhibitory contrast gain control pool, defined as:

$$G_{i}\left(c,\theta \right)=c*g_{i}\left(\theta \right)$$ (6)

where *g* captures the orientation tuning function of the gain control pool [75]. *g*_*i*_ is identical to *f*_*i*_, except that a different bandwidth parameter *k*_*inh*_ is used. The responses of neurons in the gain control pool inhibit the response of neuron *i* to the target grating, thereby causing the response *R*_*i*_ of this neuron to saturate at contrasts above *c*_50_, which is also known as the semi-saturation contrast. The average response $\bar{r_{i}}$ to the target grating (in number of spikes) is obtained by extending Eq 5 to incorporate spontaneous discharge *r*_0_ (in Hertz), maximum response rate *r*_*max*_ (in Hertz) and stimulus presentation duration *t* (in seconds):

$$\bar{r_{i}}\left(c,\theta \right)=t\left[r_{0}+r_{max}\frac{L_{i}{\left(c,\theta \right)}^{p}}{{c_{50}}^{p}+G_{i}{\left(c,\theta \right)}^{p}}\right]$$ (7)

Response variance is proportional to the average response rate [76]:

$$var\left(r_{i}\right)=\zeta \bar{r_{i}}$$ (8)

where *ζ* is a proportionality constant. Individual neural responses *r*_*i*_ are assumed to follow a normal distribution:

$$r_{i}∼\;N\left[\bar{r_{i}},var\left(r_{i}\right)\right]$$ (9)

Implementing the effect of response covariance requires elaborate Monte-Carlo simulations. To keep computations tractable, a diagonal covariance matrix is used, thereby assuming that correlations between neural responses are zero. However, V1 neurons are known to be correlated [77–80]. Note that these correlations only scale down the average signal-to-noise ratio of the population response. A lower overall signal-to-noise ratio results in lower average detection performance across all conditions. To capture such variations in overall detection performance, we included a global efficiency parameter in the model’s decoding stage (cf. infra).

Eq 7 defines the contrast response function of the standard contrast normalization model [20, 22–24]. This model is used to predict responses when AM is absent, i.e., in the Flicker condition. To account for the effects of AM, however, Eq 7 has to be extended. The average response of neuron *i* to the target grating in the presence of AM is given by:

$$\bar{r_{i}}\left(c,\theta_{tgt},\theta_{ind}\right)=t\left[r_{0}+\left[1-\gamma *h_{i}\left(\theta_{ind}\right)\right]r_{max}\frac{{\left[L_{i}\left(c,\theta_{tgt}\right)+\alpha *h_{i}\left(\theta_{ind}\right)\right]}^{p}}{c_{50}^{p}+{\left[G_{i}\left(c,\theta_{tgt}\right)+\beta *j_{i}\left(\theta_{ind}\right)\right]}^{p}}\right]$$ (10)

where *h*_*i*_ and *j*_*i*_ are identical to *f*_*i*_, except that a bandwidth parameter *k*_*exc*,*AM*_ is used for *h*_*i*_ and a bandwidth parameter *k*_*inh*,*AM*_ is used for *j*_*i*_. *θ*_*tgt*_ represents the target grating orientation and *θ*_*ind*_ equals the orientation of the two AM-inducing gratings used to create the percept of AM. We assume that AM can affect the encoding of gratings by changing the contrast response function in three major ways. (1) AM can introduce a level of excitation, controlled by *α*, (2) AM can cause inhibition, i.e., a shift of the contrast response function towards higher contrasts, controlled by *β*, and (3) AM can cause suppression, i.e., a reduction of the maximal contrast response, controlled by *γ*. In the absence of AM (as in the Flicker condition), *α*, *β* and *γ* are zero and Eq 10 reduces to Eq 7. We now discuss these effects in detail.

Via *α*, AM induces a neural response provided that the tuning function *h*_*i*_ evaluated at the inducer grating orientation is not zero. AM thus only excites neurons that are sensitive to the inducer orientation. In this way, we obtain responses “as if” the inducer was physically moving along the AM path. These responses thus reflect the “filling-in” of activation. In the special case that *θ*_*tgt*_ = *θ*_*ind*_ and *k*_*exc*_ = *k*_*exc*,*AM*_, AM will induce a response that is equal to the excitatory receptive field response that would be evoked by an inducer grating with contrast *α* presented at the target location (for instance, during a physical motion along the AM path). As the inducer gratings were presented at 100% contrast during our experiments, complete filling-in occurs when *α* equals 1. It should be noted that the tuning bandwidth parameter *k*_*exc*,*AM*_ for the AM-induced excitation was allowed to differ from the bandwidth parameter *k*_*exc*_ of the linear receptive field when fitting the model. However, the estimates of these parameters did not differ for the best-fitting model (cf. Results section).

As mentioned earlier, the responses of a given neuron *i* are normalized by the responses of other neurons in a gain control pool. By evoking responses in these gain control neurons, AM may cause divisive normalization. *β* controls the strength of this inhibitory effect. The term *β* * *j*_*i*_(*θ*_*ind*_) lowers contrast sensitivity by shifting the contrast response function to higher contrasts, but only of neurons tuned to the inducer orientation. It can be seen as an indirect effect, resulting from a more direct excitatory effect that activates the normalization mechanism. The tuning bandwidth parameter of the inhibitory effect *k*_*inh*,*AM*_ was allowed to be different from the bandwidth parameter *k*_*inh*_ of the gain control pool during fitting but again, these parameters were estimated to be equal for the best-fitting model (cf. Results section).

The third AM-induced effect is suppression through a reduction of response gain. *γ* rescales the contrast response function to lower response rates, thereby reducing the neurons’ maximal response. Similar to the other two AM effects, this suppressive effect only occurs in neurons that are tuned to the inducer orientation. The bandwidth of the tuning function is equal to the bandwidth *k*_*exc*,*AM*_ of the excitatory effect.

#### Decoding stage

In the decoding stage, neural responses are combined into decisions in our spatial 2AFC detection task. We implemented a simple linear decoder that considers the two spatial locations at which the target grating can appear. For each location, the decoder sums the responses of all neurons tuned to that location. The location yielding the largest summed response is indicated as containing the target grating. *S*_*tgt*_ equals the summed responses of those neurons tuned to the location at which the target is presented, whereas *S*_*blank*_ equals the summed responses of neurons tuned to the other location. For the AM conditions, the average values of these sums are defined as:

$$\bar{S_{tgt}}\left(c,\theta_{tgt},\theta_{ind}\right)=\sum_{i=1}^{N}\bar{r_{i}}\left(c,\theta_{tgt},\theta_{ind}\right)$$ (11)

$$\bar{S_{blank}}\left(c=0,\theta_{tgt},\theta_{ind}\right)=\sum_{i=1}^{N}\bar{r_{i}}\left(\theta_{tgt},\theta_{ind}\right)$$ (12)

where $\bar{r_{i}}$ is provided by Eq 10. For the Flicker condition, $\bar{S_{tgt}}$ and $\bar{S_{blank}}$ are obtained in a similar fashion, but using the standard contrast response function of Eq 7 instead of the elaborated version of Eq 10. In agreement with Vogels et al. [76], the variance of the summed responses is provided by:

$$var\left(S_{tgt}\right)=\zeta \bar{S_{tgt}}$$ (13)

and

$$var\left(S_{blank}\right)=\zeta \bar{S_{blank}}$$ (14)

Proportion correct detection *p* in our task is then provided by the cumulative Gaussian function:

$$p=\int_{0}^{+\infty }\frac{1}{\sigma \sqrt{2\pi }}e^{\frac{-{\left(x-\mu \right)}^{2}}{2\sigma^{2}}}dx$$ (15)

with

$$\mu =\bar{S_{tgt}}-\bar{S_{blank}}$$ (16)

and

$$\sigma =\frac{1}{\epsilon }\sqrt{var\left(S_{tgt}\right)+var\left(S_{blank}\right)}$$ (17)

where *ϵ* is an efficiency parameter. This parameter can accommodate the effect of interneural correlation as mentioned previously, but can also capture other factors that may affect overall performance such as global attention level and fatigue. The purpose of our study is not to distinguish between these factors, as they only affect absolute performance and do not cause relative differences in performance between AM and Flicker conditions.

It should be noted that our decoder does not use a-priori knowledge of the target grating orientation when summing filter responses. A more optimal decoder may preferentially weight filters that are tuned to the grating while ignoring filters tuned to other, irrelevant orientations. Such a decoder is not plausible in our experiments, however, as multiple target orientations were randomized across trials. Observers did not know the target orientation at the start of each trial. It would be impossible for them to implement a detection strategy tailored to grating orientation without first detecting the grating. In addition, the same decoding strategy is used in the AM and Flicker conditions. In other words, the decoder does not account for the effects of AM on filter responses. The fact that we observe strong masking indeed suggests that the decoder does not manage to discount or compensate for the AM-induced effects on the population response.

Previous studies have shown that observers are to some extent uncertain about the exact spatial location of the target in grating detection tasks, which increases the slope of the psychometric function [35]. This spatial uncertainty effect could be captured by assuming a more complex decoder in our model, for instance, a non-linear decoder that selects the maximum of all neural responses to obtain the decision variable instead of computing a linear sum [35]. Implementing such a decoder would involve elaborate Monte-Carlo simulations as the distribution of the decision statistic cannot be obtained analytically (cf. Eqs 11 and 13). Furthermore, the population of neurons assumed in our model would have to be expanded considerably to include subpopulations that are sensitive to irrelevant locations. As these operations would render model fitting computationally prohibitive, we did not implement non-linear decoding. This does not imply that our model cannot capture the increased slope of the psychometric function in the presence of spatial uncertainty. An increase of the response exponent *p* allows for such an increased slope. Consequently, the estimated value of *p* should not be taken to solely reflect V1 response acceleration but may also capture other factors mediating psychometric function steepness. Separating these factors is not a goal of the present study, as we show in the Results section that psychometric function steepness is unrelated to AM.

### Model constraints and fitting

Three parameters were poorly constrained by our data and were fixed to physiologically plausible values. These values can be changed without affecting the conclusions of this study. The concentration parameter of the gain control tuning function *k*_*inh*_ was set to 0.001, resulting in a broadly tuned gain control pool [55]. *r*_*max*_ was fixed at 100 Hz [81]. *ζ*, the proportionality constant controlling response variance, was fixed at 1.9 [26, 76]. A number of additional constraints were introduced. As physiological studies suggest rather low spontaneous discharge rates at the level of V1, *r*_0_ was constrained to be smaller than 5% of the maximal response *r*_*max*_. The tuning functions controlling the orientation selectivity of the AM effects were not allowed to be narrower than the orientation tuning functions of the excitatory receptive fields. The reason for this constraint is that effects induced by AM are presumably the result of feedback from higher visual areas specialized in motion, such as hMT/V5+ [4–6]. These areas are typically characterized by a lower orientation selectivity compared to V1 [29]. It is therefore unlikely that the AM effects are more selective to orientation than V1 cells. The response exponent *p* was not allowed to be smaller than 2 [28] and the efficiency parameter *ϵ* was constrained between 0% and 100%.

A total of 10 parameters were estimated using a maximum-likelihood fitting procedure [69]. Multiple fits were performed using randomized starting values for each parameter. Akaike’s Information Criterion (AIC) was calculated to assess the goodness-of-fit of the models while taking into account the complexity of the model quantified as the number of fitted parameters. Parametric Monte-Carlo bootstrapping involving 1000 samples provided the confidence intervals of the estimated parameters and the distributions of the AIC statistics which were used in evaluating the quality of the model fit [70].

## Supporting Information

S1 Text Individual data and pooling. (PDF)

S2 Text Control experiment measuring eye movements during the detection task. (PDF)
