# Supplementary material for: Apparent Motion Suppresses Responses in Early Visual Cortex: A Population Code Model
Source: PLoS Comput Biol. 2016 Oct 26;12(10):e1005155. doi: 10.1371/journal.pcbi.1005155 (PMC5081194; doi:10.1371/journal.pcbi.1005155)
Supplement: S1 Text — (PDF) [file pcbi.1005155.s001.pdf]

Supporting Information

S1 Text. Individual data and pooling

Each observer exhibited decreased detection performance in the AM condition compared to the Flicker condition. In addition, the AM masking was orientation tuned for each observer. Figs. S1-S5 show the individual data sets for each observer. The best-fitting psychometric functions are denoted by dashed lines, while the best-fitting population code models are depicted by full lines.

All observers showed significantly reduced maximal performance $(1 - \lambda)$ compared to the Flicker condition. For each observer except SG, the fit of the full population code model was significantly better than a reduced model including only excitation and inhibition (AIC full model - AIC restricted model < 0, parametric bootstrap, $p < 0.05$ after Bonferroni correction). For observer SG, the model including suppression still outperforms the reduced model (AIC difference = -20.07). The AM-induced suppression was also significant for this observer ($\gamma = 53.36\%$, parametric bootstrap, $95\% CI = [41.22\%, 69.23\%]$). All observers displayed significant orientation tuning of AM masking. A model assuming no tuning of AM effects (i.e., $k_{exc,AM}$ and $k_{inh,AM}$ are fixed at 0.001) provided a significantly worse fit than our full model for all observers (AIC full model - AIC restricted model < 0, parametric bootstrap, $p < 0.05$ after Bonferroni correction). As patterns were highly similar across observers, the data were normalized and pooled using the following procedure [1]. For each observer and condition (AM or Flicker condition), we estimated the position c_m of the psychometric function along the contrast axis as well as the level of psychometric function compression λ. Within each condition, we divided all contrasts by c_m and multiplied them by the value of c_m averaged across all observers and conditions. Performance levels p were normalized using the following formula:

$$p_{norm} = 0.5 + (p - 0.5)\,\frac{1 - \lambda_{avg}}{1 - \lambda} \qquad (1)$$

where p_{norm} is the normalized performance and λ the compression level for a specific observer and condition. λ_{avg} is the value of λ averaged across observers and conditions. After normalization, data were pooled to obtain a single data set representing the average performance of all observers.

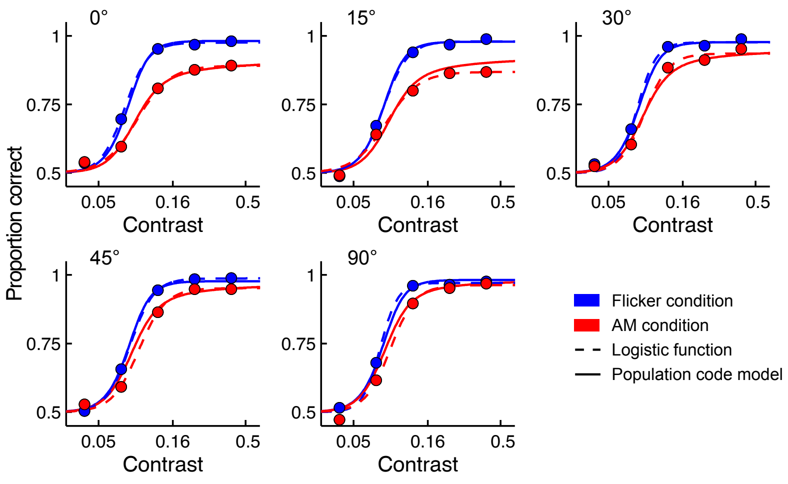

Fig S1. Model fits to data of observer SG. Plots are shown for each orientation difference between the target and inducer gratings. Red and blue symbols denote the AM and Flicker conditions respectively. Dashed lines depict the best-fitting logistic psychometric functions, while full lines represent the best-fitting population code model.

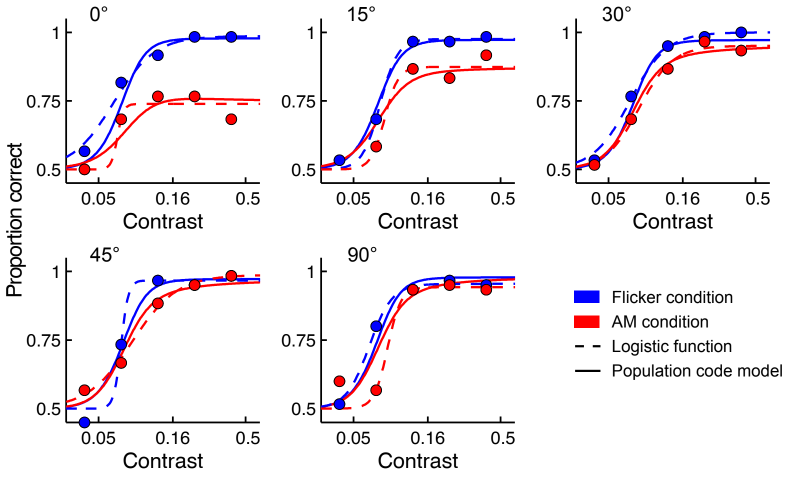

Fig S2. Model fits to data of observer BO. Plots are shown for each orientation difference between the target and inducer gratings. Red and blue symbols denote the AM and Flicker conditions respectively. Dashed lines depict the best-fitting logistic psychometric functions, while full lines represent the best-fitting population code model.

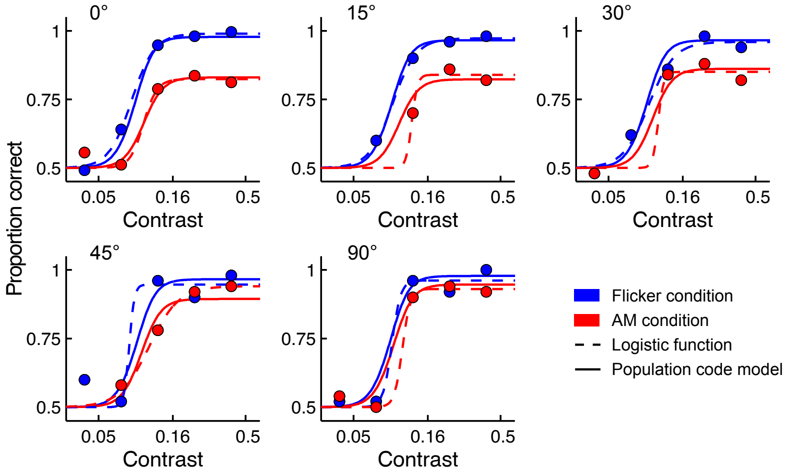

Fig S3. Model fits to data of observer EV. Plots are shown for each orientation difference between the target and inducer gratings. Red and blue symbols denote the AM and Flicker conditions respectively. Dashed lines depict the best-fitting logistic psychometric functions, while full lines represent the best-fitting population code model.

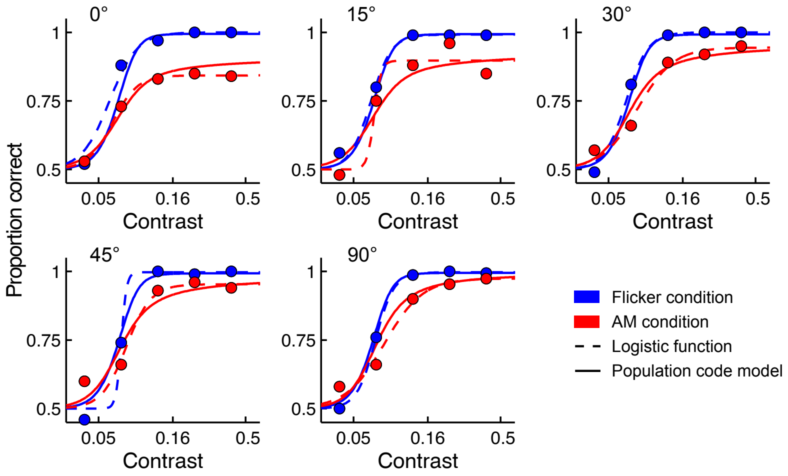

Fig S4. Model fits to data of observer AV. Plots are shown for each orientation difference between the target and inducer gratings. Red and blue symbols denote the AM and Flicker conditions respectively. Dashed lines depict the best-fitting logistic psychometric functions, while full lines represent the best-fitting population code model.

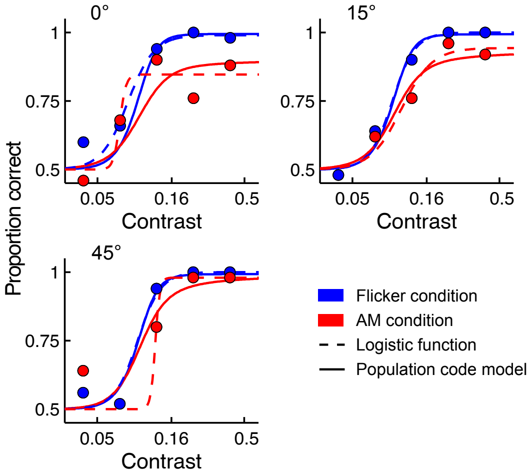

Fig S5. Model fits to data of observer CV. Plots are shown for each orientation difference between the target and inducer gratings (no data was collected for this observer for orientation differences of 30° and 90°). Red and blue symbols denote the AM and Flicker conditions respectively. Dashed lines depict the best-fitting logistic psychometric functions, while full lines represent the best-fitting population code model.

References

1. Putzeys T, Bethge M, Wichmann F, Wagemans J, Goris R. A new perceptual bias reveals suboptimal population decoding of sensory responses. PLoS Comput Biol. 2012;8(4):e1002453.
