# Supplementary material for: Apparent Motion Suppresses Responses in Early Visual Cortex: A Population Code Model
Source: PLoS Comput Biol. 2016 Oct 26;12(10):e1005155. doi: 10.1371/journal.pcbi.1005155 (PMC5081194; doi:10.1371/journal.pcbi.1005155)
Supplement: S2 Text — (PDF) [file pcbi.1005155.s002.pdf]

Supporting Information

S2 Text. Control experiment measuring eye movements during the detection task

A control experiment was conducted to examine whether observers' eye positions remained fixated on the fixation cross during the course of a trial. The stimuli and design were identical to those used in the main experiment, except that eye movements were continuously recorded (see Methods) and that the orientation of the target was always horizontal, i.e., equal to the orientation of the AM-inducing stimuli. Data were collected for three observers (BV, FF, and JM), who were naive to the purpose of the experiment. The experiment consisted of blocks of 50 trials, in which contrast (5) and condition (2) levels were randomised. Each subject completed at least 100 trials for each combination of these levels, yielding a total of 1000 trials per subject.

Observers were found to be highly successful in maintaining fixation on the fixation cross throughout the course of a trial. Fig. S6 shows a heatmap containing all fixation locations for the three observers. It can be seen that observers' fixations are concentrated in a small region surrounding the centre of the display. 95% of deviations in the x and y direction are within a radius of approximately 1° from the fixation cross.

To determine whether eye movements played a role in AM masking, we compared the average fixation location in the AM versus Flicker condition along both the horizontal and vertical axis. We found no significant difference between the AM and Flicker condition for two observers (BO and FF). Fig. S7 provides histograms of the x and y coordinates of fixation locations for observer FF in the Flicker (top row) and AM condition (bottom row). For observer JM however, the difference along the vertical axis between the Flicker and AM condition was significant (difference = .054°, non-parametric bootstrap, $p < 0.001$). This observer fixated on average at a vertical position of 0.195° above the center of the screen in the AM condition, as opposed to 0.141° in the Flicker condition.The average fixation location along the horizontal axis was not significantly different between the AM and Flicker condition for this observer (non-parametric bootstrap, $p = 0.84$).

The average fixation location measure does not provide information about the amplitude of eye movements. Indeed, large eye movements away from the screen center in opposite directions may partially cancel out in the calculation, leading to an average fixation location close to the screen center. For this reason, we also analysed the average eye movement amplitude, computed as the absolute value of difference between each fixation location and the center of the screen. Again, no significant difference in average amplitude between AM and Flicker conditions was observed for subjects BO and FF. Observer JM, on the other hand, showed an average amplitude in the horizontal direction of 0.403° in the AM condition, which was 0.08° larger than the average horizontal amplitude in the Flicker condition. The vertical amplitude in the AM condition was 0.304°, which was 0.034° larger than the corresponding amplitude in the Flicker condition. Both these differences were significant (non-parametric bootstrap, $p < 0.001$).

The observed differences in average fixation position and eye movement amplitude for observer JM cannot account for the differences in detection performance between the AM and Flicker condition. The reason is that the size of the differences is small compared to the size of typical V1 receptive fields, which can be as large as 2° for large

eccentricities [1]. This implies that the majority of V1 receptive fields that are stimulated in the AM condition will also be stimulated in the Flicker condition. The only difference is that the visual image will be slightly spatially shifted relative to the receptive field. Such a shift is comparable to a shift in the spatial phase of the inducer and target gratings. In this respect, it should be noted that Hidaka et al. [2] found that AM masking does not depend on spatial phase. Presumably, neurons involved in AM masking are phase-insensitive (e.g., V1 complex cells). Consequently, spatial position shifts due to eye movements cannot account for the AM masking observed in our study. Likewise, these shifts cannot explain the observed orientation tuning of AM masking in the main experiment, as it is highly unlikely that the small difference in average amplitude of vertical and horizontal eye movements between the Flicker and AM condition would result in degraded detection performance for a horizontal target grating in the AM condition.

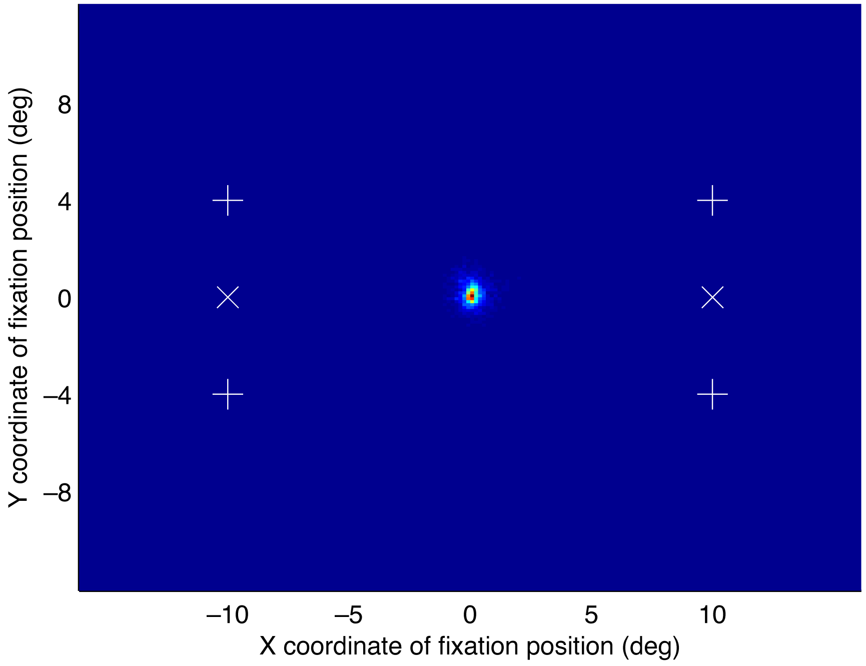

Fig S6. Heatmap indicating the fixation locations of all observers measured during the control experiment. Blue and red color respectively represent a low and high density of fixations. The possible positions of the target grating and the AM-inducing stimuli are respectively denoted by "x" and "+" symbols.

Fig. S8 shows the contrast detection data set for a representative observer (FF). As in the main experiment, we found AM-induced masking. Our population code model was fitted to the data of each observer individually. The model was able to account for the data of all observers using parameter values that were highly similar to the original estimates obtained for the main experiment: we were able to constrain 8 of the 10 free parameters to the original estimates. Only the efficiency parameter ϵ and the response exponent p were adjusted to fit the data of the control experiment. The resulting AIC value is significantly lower than the AIC of the original population code model (average AIC difference = 238.34, parametric bootstrap, $p < 0.001$ for all observers), suggesting that the model fit to the control experiment is acceptable and better than the original

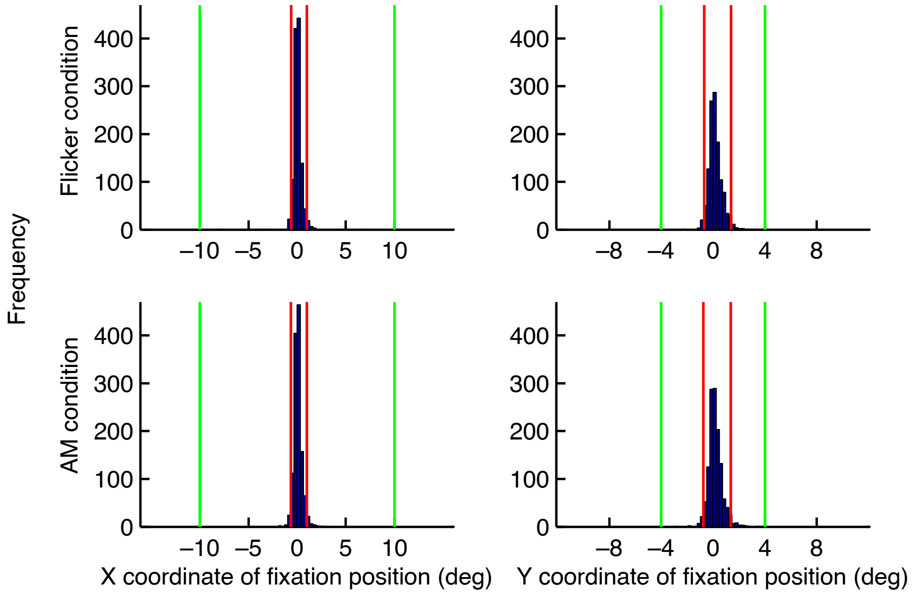

Fig S7. Fixation locations of observer FF measured during the control experiment. Histograms are shown for the x (left) and y (right) coordinates of the fixation locations observed for observer FF for the Flicker (top row) and AM condition (bottom row). Red vertical lines denote the 95% confidence intervals. The x coordinates for the possible positions of the target grating and the y coordinates for the possible positions of the AM-inducing stimuli are depicted by green vertical lines.

fit when considering the lower model complexity resulting from the lower number of free parameters. The model prediction is denoted by the full lines in Fig. S8.

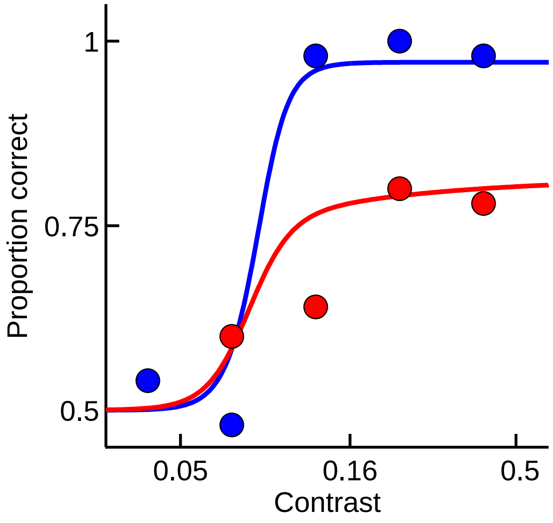

Fig S8. Model fit to data of observer FF in the control experiment. Red and blue symbols denote the AM and Flicker conditions respectively. Full lines depict the predictions of the best-fitting population code model.

The efficiency parameter and the response exponent were respectively estimated at 76% ($SEM = 12.23\%$) and 5.56 ($SEM = 1.45$) on average across observers. These averages are close to the original estimates found for the main experiment. This is due to the fact that only the efficiency parameter of observer BV ($\epsilon = 100\%$) and the response exponent of observer JM ($p = 2.98$) differed significantly (parametric bootstrap, $p < 0.05$ after Bonferroni correction). A difference in the efficiency parameter is not unexpected, as this parameter is able to capture the effects of a wide range of factors. Likewise, the response exponent may not only reflect V1 response acceleration (which is presumably stable across observers and experiments), but also spatial uncertainty as argued in the main text. It is possible that the complete absence of large eye movements led to a reduction of spatial uncertainty for observer JM, thereby lowering the response exponent.

The fact that the control experiment data can be predicted nearly blindly, i.e., by returning only two specific parameter estimates from the main experiment data, supports the validity of our model. It also strongly suggests that the AM-induced effects captured by the model are not due to eye movements. Indeed, the values of the parameters α, β and γ controlling these effects were fixed and thus identical in the main and control experiment. For this control experiment, the contrast detection data of observer JM did not differ from the data of the two observers that did not show significant differences in eye movements, as all data could be fit using the same model.

References

1. Cavanaugh JR, Bair W, Movshon JA. Nature and interaction of signals from the receptive field center and surround in macaque V1 neurons. J Neurophysiol. 2002;88(5):2530–2546.

2. Hidaka S, Nagai M, Sekuler AB, Bennett PJ, Gyoba J. Inhibition of target detection in apparent motion trajectory. J Vis. 2011;11(10):1–12.
